# Expression of the pacemaker channel HCN4 in excitatory interneurons in the dorsal horn of the murine spinal cord

**DOI:** 10.1186/s13041-020-00666-6

**Published:** 2020-09-18

**Authors:** Taku Nakagawa, Toshiharu Yasaka, Noriyuki Nakashima, Mitsue Takeya, Kensuke Oshita, Makoto Tsuda, Ken Yamaura, Makoto Takano

**Affiliations:** 1 Department of Physiology, Kurume University School of Medicine, 67 Asahi-Machi, Kurume, 830-0011 Japan; 2 Department of Anesthesiology, Graduate School of Medicine, Kyushu University, Fukuoka, 812-8582 Japan; 3 Department of Health and Nutrition, Niigata University of Hearth and Welfare, Niigata, 950-3198 Japan; 4 Department of Anesthesiology, Kurume University School of Medicine, Kurume, 830-0011 Japan; 5 Department of Life Innovation, Graduate School of Pharmaceutical Sciences, Kyushu University, Fukuoka, 812-8582 Japan

**Keywords:** Hyperpolarization-activated cyclic nucleotide-gated channels, Spinal cord, Immunohistochemistry, Protein kinase Cγ, Parvalbumin, Glutamate decarboxylase 67, Vesicular GABA transporter, Vesicular glutamate transport protein 2, Tactile allodynia

## Abstract

In the central nervous system, hyperpolarization-activated, cyclic nucleotide-gated (HCN1–4) channels have been implicated in neuronal excitability and synaptic transmission. It has been reported that HCN channels are expressed in the spinal cord, but knowledge about their physiological roles, as well as their distribution profiles, appear to be limited. We generated a transgenic mouse in which the expression of HCN4 can be reversibly knocked down using a genetic tetracycline-dependent switch and conducted genetically validated immunohistochemistry for HCN4. We found that the somata of HCN4-immunoreactive (IR) cells were largely restricted to the ventral part of the inner lamina II and lamina III. Many of these cells were either parvalbumin- or protein kinase Cγ (PKCγ)-IR. By using two different mouse strains in which reporters are expressed only in inhibitory neurons, we determined that the vast majority of HCN4-IR cells were excitatory neurons. Mechanical and thermal noxious stimulation did not induce c-Fos expression in HCN4-IR cells. PKCγ-neurons in this area are known to play a pivotal role in the polysynaptic pathway between tactile afferents and nociceptive projection cells that contributes to tactile allodynia. Therefore, pharmacological and/or genetic manipulations of HCN4-expressing neurons may provide a novel therapeutic strategy for the pain relief of tactile allodynia.

## Introduction

Hyperpolarization-activated, cyclic nucleotide-gated (HCN) channels are expressed in both the peripheral nervous system (PNS) and central nervous system (CNS), and these ion channels generate the hyperpolarization-activated, non-selective cation current (I_h_). The HCN channel family comprises four subtypes (HCN1–4) that form heteromultimers. Among those, HCN1 and HCN4 possess the fastest and slowest activation kinetics upon hyperpolarization, respectively. The voltage-dependence of activation of HCN2 and HCN4 is highly sensitive to cyclic adenosine monophosphate (cAMP), whereas that of HCN1 and HCN3 is almost insensitive to cAMP [1].

In the PNS, HCN1 and HCN2 are the major isoforms expressed in the dorsal root ganglion (DRG). HCN4, a well-known pacemaker channel in the sinoatrial node, is expressed in a minor population of trigeminal ganglion and DRG neurons [2]. Most notably, HCN2 channels expressed in small-diameter DRG neurons reportedly play a pivotal role in inflammatory nociception [3–5]. In the CNS, HCN1 and HCN2 are broadly expressed in the cerebral cortex, cerebellar cortex, basal ganglia, and hippocampus [6, 7]. HCN4 is reportedly expressed in olfactory and thalamocortical neurons [8–10]. In these loci, HCN channels are proposed to regulate the resting membrane potential, dendritic integration of synaptic potentials, repetitive firing, and oscillatory activities in neural circuits [1, 10].

In the spinal cord, HCN channels are reportedly expressed in both the ventral and dorsal horns [11–14]. While some afferent information from the PNS is directly transmitted to projection neurons that have axons traveling to higher CNS regions, most of it is processed by the network of interneurons in the spinal dorsal horn (SDH) and then transmitted to projection neurons [15, 16]. It is, therefore, anticipated that HCN channels may play important roles in the regulation of synaptic transmission in local circuits of the SDH. Although it has been reported that HCN4 is the most frequent HCN subtype in the SDH, the neurotransmitter phenotype of these neurons remains unclear. HCN4-immunoreactivities are found in parvalbumin (PV) and protein kinase Cγ (PKCγ) neurons [17]. While PKCγ is expressed in a subset of excitatory neurons, PV is shared by subsets of excitatory and inhibitory neurons. It has been suggested that putative inhibitory PV cells express HCN4 because these cells present pronounced I_h_ currents and high-frequency firing [17]. On the other hand, HCN4 is not likely colocalized with Pax2, a marker of inhibitory neurons, but is localized on presynaptic terminals of glutamatergic interneurons in the SDH [14]. In the present study, we carried out immunohistochemistry using double transgenic mice in which the expression level of HCN4 could be reversibly knocked down by doxycycline administration. With the aid of genetic validation for the locus of HCN4-immunoreactivity, we found that the majority of HCN4-immunoreactivity was localized in excitatory interneurons of laminae II–III.

## Methods

### Ethical approval

All animal experiments in this report were approved by the Animal Ethics Committee of Kurume University animal care and experiments (#2020–30). Animal care and experiments conformed to the Guidelines for the Care and Use of Laboratory Animals published by the US National Institutes of Health (NIH Publication No. 85–23, revised 1996).

### Animals

The generation of the HCN4^+/tTA_TRE^ mice has been described previously [8, 18]. HCN4^+/tTA_TRE^ mice were identified by genomic PCR using the following primers: forward (5′-CGA ATA AGA AGG CTG GCT CTG CAC C-3′), reverse (5′-GAG CAG CCT ACA TTG TAT TGG CAT G-3′). Inhibition of HCN4 expression by the mutant allele was performed by providing food pellets containing doxycycline (DOX; 6 mg g^− 1^, Research Diet, NJ, USA) ad libitum for 2 weeks before the experiments [19]. HCN4^+/Luc^ mice were generated as previously reported [19]. HCN4^+/Luc^ mice were identified by genomic PCR using the following primers: forward (5′-CGA GCT GGA CGG CGA CGT AAA CGG C-3′), reverse (5′-CAT CCT TAG GGA GAA TTT GTT GAC C-3′). The knock-in mice in which cDNA of GFP had been targeted into the locus of glutamate decarboxylase 67 (GAD67-GFP mouse) were obtained from RIKEN BRC (#03674) [20]. These GAD67-GFP mice were identified by genomic PCR using the following primers: forward (5′-CCT TCT GTC TCC GAG ACT TCG GTG-3′), reverse (5′-TCC TTG AAG AAG ATG GTG CG-3′). The vesicular gamma-aminobutyric acid (GABA) transporter (VGAT)-mutant YFP (Venus) bacterial artificial chromosome (BAC) transgenic mouse (VGAT-Venus mouse) was obtained from RIKEN BRC (#09645) [21]. The VGAT-Venus mice were identified by genomic PCR using the following primers: forward (5′-CGC TCA CCT TGG CCT GGG ACT TGT T-3′), reverse (5′-TGA GCT ACC AGT CCG CCC TGA GCA A-3′). The mice were housed in groups of six per cage in the same room under 12/12 h light/dark cycle at 25 °C and 60% humidity. Water and food pellets were provided ad libitum. In this study, 16- to 24-week-old mice of both sexes were deeply anesthetized with urethane (1.5 g kg^− 1^) and then used for the following experiments.

### Luminescence imaging

150 μL of XenoLight RediJect D-luciferin (30 mg mL^− 1^; PerkinElmer, Waltham, MA, USA) was injected intraperitoneally into HCN4^Luc/tTA_TRE^ mice. The mouse was deeply anesthetized 30 min after the injection, and the lumbar spinal cord was dissected out. Transverse sections (400 μm thickness) of the spinal cord were obtained using a microslicer (Linear Slicer PRO 1; Dosaka EM, Kyoto, Japan). Luminescent imaging was performed using an EM-CCD camera (ImagEM2; Hamamatsu Photonics, Hamamatsu, Japan).

### Quantitative PCR

After deep anesthesia, the mice were perfused transcardially with RNAlater (Ambion, Grand Island, New York, USA). Thereafter, lumbar spinal cords were dissected out and stored in the same solution. Horizontal sections of L3-L4 (600 μm thickness) were obtained using a microslicer. Total RNA was extracted from the preparation using Trizol reagent (Thermo Fisher Scientific, Waltham, MA, USA). Superscript III (Thermo Fisher Scientific) was used to synthesize single-strand cDNA. We used predesigned TaqMan Gene Expression assay probes for mouse HCN4 (Mm01176086_m1; Thermo Fisher Scientific) to perform the quantitative real-time polymerase chain reaction (qPCR). Relative levels of mRNAs were normalized to the level of 18S rRNA (4319413E; Thermo Fisher Scientific).

### Fixation and immunohistochemistry

After deep anesthesia, the mice were perfused transcardially with 4% paraformaldehyde solution. The lumbar spinal cords were dissected out and post-fixed in the same fixative for 2 h. Then, the spinal cords were cryoprotected overnight in phosphate-buffered saline containing 30% w/v sucrose and mounted in OCT Compound (Sakura Finetek, Tokyo, Japan). Transverse sections (30 μm thickness) were cut on a cryostat (CM3050S; Leica Microsystems, Wetzlar, Germany), incubated in 50% ethanol for 30 min to enhance antibody penetration, and then incubated for 72 h in a cocktail of primary antibodies (see Additional file 1, Supplementary Table S1). Immunolabeling of primary antibodies was visualized using species-specific secondary antibodies conjugated to DyLight 405, Alexa 488, Alexa 594, and Alexa 647 (Additional file 1, Supplementary Table S2). Representative sections of the spinal cord from at least three animals for each examination were scanned with a confocal microscope (FV1000; Olympus, Tokyo, Japan; laser wavelengths = 405 nm, 473 nm, 559 nm, and 635 nm). The scanned images were processed using FV10-ASW software (Olympus). In the present study, we defined an HCN4-immunoreactive (IR) cell as a cell that showed circumferential membrane immunostaining. NeuN immunostaining was used to mark the nucleus of a neuron. We also defined the dense plexus of PKCγ-IR cells as the ventral part of the inner lamina II (II_iv_). The lamina III was defined as the gray matter that has 80 μm thickness under lamina II, according to the Allen Brain Atlas [22].

### Quantitative analysis of spinal dorsal horn cells

To examine the proportion of neurons in laminae I–III, we scanned the dorsal horn sections sequentially with a 20× lens and produced z-series of 8 optical sections in 2-μm steps. It was necessary to scan 1–2 z-series from each section to cover the whole area of laminae I–III. We first determined the gray matter outlines and then the boundaries between the dorsal part of the inner lamina II (II_id_), II_iv_, and lamina III, referring to the immunoreactivity of the PKCγ plexus. The ventral border of lamina III was drawn according to the definition of lamina III as mentioned above. We initially marked the cell nuclei with NeuN whose sizes were largest in the 3rd to 6th sections of each z-series consisting of 8 sections. We then examined the HCN4-immunoreactivity of the marked cells. Coexpression of GAD67-GFP or VGAT-Venus fluorescence was assessed in the same way. We did not perform NeuN immunohistochemistry when we examined the colocalization of PKCγ-, PV-, or c-Fos-immunoreactivities with HCN4-immunoreactivity.

### Noxious stimulation

Six HCN4^Luc/tTA_TRE^ mice were used in these experiments. After the mice were deeply anesthetized, we applied one of the following two types of noxious stimuli: (1) pinching of skin folds at 10 points by the same experimenter in the same way (5 each on dorsal and ventral surfaces of the left hind paw, *n* = 3 mice). Each point of the skin was pinched using Adson forceps with 1 × 2 teeth (1.5 mm tips, World Precision Instruments #500092) for 3 s with equal force until the teeth of forceps completely bite into the skin: (2) immersion of the left hind paw in water at 52 °C for 15 s (*n* = 3 mice). Two hours after the noxious stimulation, the mice were transcardially perfused with 4% paraformaldehyde under urethane anesthesia (1.5 g kg^− 1^). Thereafter, the lumbar spinal cords of these mice were dissected out and used for immunohistochemistry and quantitative analysis, as described above.

### von Frey assay

We carried out von Frey assay using wild type mice (*n* = 3) and HCN4^Luc/tTA_TRE^ mice which were fed with DOX-containing food pellet for 4 weeks (*n* = 3). These mice were placed on an elevated grid of wire and the plantar surface of hind paw was stimulated with calibrated von Frey monofilaments (0.04–2 g, Muromachi, Tokyo, Japan). Fifty percent paw withdrawal threshold was determined by Dixon’s up-down method [23]. In each mouse, the threshold value was averaged from those measured in bilateral hind paws.

### Data analysis

**Fig. 1:**
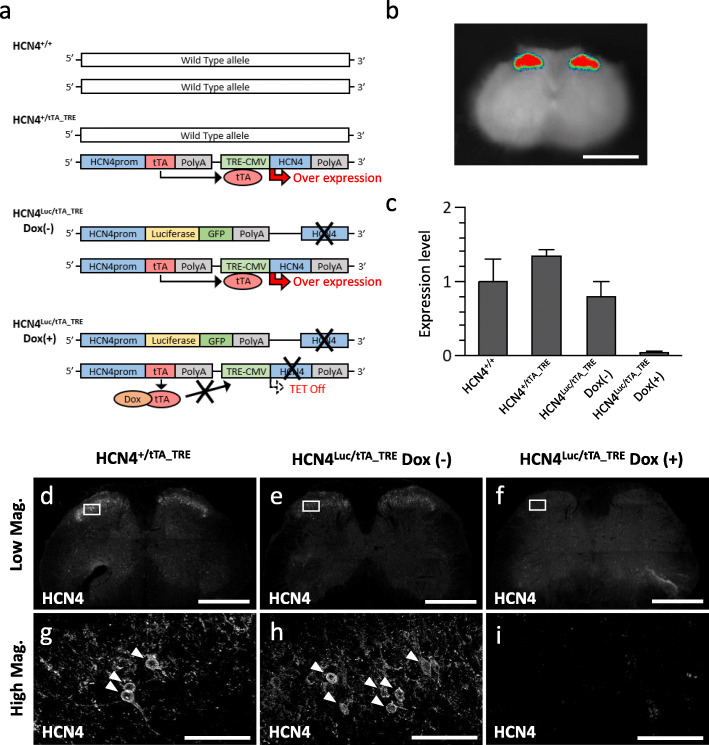
Luminescence imaging and genetic validation of HCN4-immunoreactivity in HCN4 conditional knockdown mice. **a** Schematic diagram representing the genetic constructs of transgenic mice used in this study. From top to bottom: HCN4^+/+^ (wild-type mouse), HCN4^+/tTA_TRE^ (tetracycline-dependent heterozygous HCN knock-in mouse), HCN4^Luc/tTA_TRE^ DOX(−) (firefly luciferase cDNA and tetracycline-dependent genetic switch double knock-in mouse without doxycycline [DOX] treatment), and HCN4^Luc/tTA_TRE^ DOX(+) (the same double knock-in mouse with DOX treatment). tTA: tetracycline transactivator; TRE-CMV: tetracycline-responsive element fused with a cytomegalovirus minimal promoter. **b** Ex-vivo luminescence imaging of a transverse lumbar spinal cord section from an HCN4^Luc/tTA_TRE^ mouse. The luminescence intensity is shown as a pseudocolor spectrum. Scale bar, 1 mm. **c** qPCR analysis of HCN4 mRNA from the transgenic mice shown in **a**. DOX was administered for 2 weeks. The expression levels of HCN4 relative to 18S rRNA were normalized to that in wild-type mice. * *P* < 0.03, ** *P* < 0.01. **d-i** Transverse spinal sections transgenic mice. **d**, **g** HCN4^+/tTA_TRE^ mouse. **e**, **h** HCN4^Luc/tTA_TRE^ DOX(−) mouse. **f**, **i** HCN4^Luc/tTA_TRE^ DOX(+) mouse. Upper and lower panels are images at low and high magnifications, respectively. In **g** and **h**, arrowheads indicate cells showing circumferential immunostaining. Note that both circumferential and punctate immunostainings are not visible in **i**. Scale bars, 200 μm (**d**-**f**) and 50 μm (**g**- **i**)

For multiple comparisons in Fig. 1c, the one-way ANOVA followed by Tukey’s test was used. Differences were considered statistically significant with *P* < 0.05. The statistical analysis was performed using KaleidaGraph (Synergy Software).

## Results

### Visualization of HCN4-expressing loci and genetic validation of HCN4-antibody specificity in the spinal cord

We have previously demonstrated that HCN4-expressing loci in the heart can be visualized with chemical luminescence using the HCN4^+/Luc^ mouse [19]. In this knock-in mouse, we inserted the cDNA encoding the fusion protein of firefly luciferase and GFP at the translation initiation site of HCN4. As shown in Fig. 1b, robust luciferase luminescence was restricted to the dorsal horn of the lumbar spinal cord. No significant luminescent signals were detected in the ventral horn. At the single-cell level, however, it was difficult to identify the precise locus of the luminescence signal due to light scattering. The fluorescent signal derived from GFP fused with the C-terminus of the luciferase was weak and indistinguishable from the background fluorescence.

To overcome this problem, we aimed to conduct the immunohistochemical study with genetic validation. Previously, we developed the HCN4^+/tTA_TRE^ mouse [8]. In the mutant allele of this HCN4^+/tTA_TRE^ mouse, the HCN4 promoter drives the tetracycline transactivator (tTA) expression, which then binds to the tetracycline responsive element followed by a cytomegalovirus minimal promoter, and overexpresses the HCN4 protein in physiological expression loci (Fig. 1a). We then crossed HCN4^+/Luc^ and HCN4^+/tTA_TRE^ mice to generate HCN4^Luc/tTA_TRE^ mice. In this double knock-in mouse, the expression of HCN4 could be completely suppressed by DOX administration, as demonstrated by the qPCR experiments in Fig. 1c. The HCN4 expression level in HCN4^+/tTA_TRE^ mice was 1.4 times higher than that of wild-type mice, but this was not statistically different (*P* = 0.17, one-way ANOVA, *n* = 3 mice).

After confirming that the conditional knockdown of HCN4 expression had been successful, we carried out immunohistochemical studies in HCN4^+/tTA_TRE^ mice (Fig. 1d, g) and HCN4^Luc/tTA_TRE^ mice without (Fig. 1e, h), and with (Fig. 1f, i) DOX administration. It was evident from these experiments, that the HCN4-immunoreactivity showed a laminar distribution in the SDH. This finding agreed well with the distribution of chemical luminescence shown in Fig. 1b. It should be also noted that the intensities of HCN4-immunoreactivity in the three types of transgenic mice were in parallel with those of mRNA levels shown in Fig. 1c. Most notably, no apparent HCN4-immunoreactivity was detected in HCN4^Luc/tTA_TRE^ mice with DOX administration (Fig. 1f, i), proving that the antibody used provides specific immunoreactivity against HCN4. At higher magnification, HCN4-immunoreactivity localized at the cell membrane of the soma showing circumferential fluorescent signals. HCN4-immunoreactivity also showed a punctate staining pattern indicating that HCN4 was expressed on neurites or axon terminals. These findings were in line with those in previous reports [13, 14, 17].

### Laminar distribution of HCN4-IR cells

**Fig. 2:**
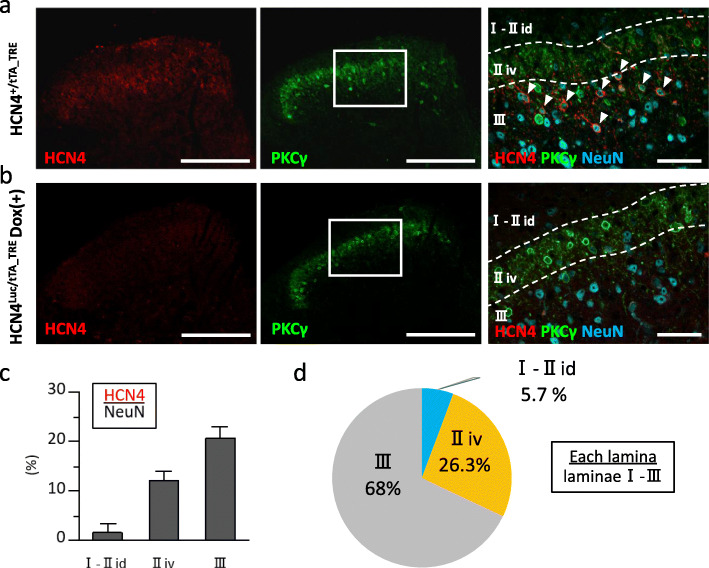
Laminar distribution of HCN4-IR cells. **a** Immunohistochemistry of the SDH in an HCN4^+/tTA_TRE^ mouse. *Left panel*, HCN4 immunoreactivity (red). *Center panel*, PKCγ immunoreactivity (green). *Right panel*, high-magnification image expanding the white box of the *center panel*. Overlay of HCN4 (red), PKCγ (green), and NeuN (cyan) IR signals. HCN4-IR cells are indicated by arrowheads. Dashed lines indicate the borders of the PKCγ plexus (upper line, border between lamina II_id_ and lamina II_iv_; lower line, border between lamina II_iv_ and lamina III). **b** Immunohistochemistry of the SDH in an HCN4^Luc/tTA_TRE^ DOX(+) mouse. *Left panel*, no HCN4-IR cells can be identified. *Center panel,* the pattern of PKCγ-immunoreactivity is essentially the same as in **a**. *Right panel*, colors and lines correspond to those in **a**. Note that there are no HCN4-IR cells. **a**, **b** Scale bars, 200 μm (*left and center panels*), 40 μm (*right panel*). **c** Percentages of HCN4-IR cells per NeuN-IR cells in each lamina. **d** Proportion of HCN4-IR cells per each lamina I–III. The ratio was calculated as the number of HCN4-IR cells in a layer divided by the total number of HCN4-IR cells in laminae I–III

Next, we compared the laminar distribution of HCN4-IR cells with that of the plexus of PKCγ-IR cells, which is widely used in mice as the marker of lamina II_iv_ (Fig. 2) [24]. In this experiment, we defined an HCN4-IR cell as a cell with circumferential membrane immunostaining and determined the proportion of HCN4-IR to NeuN-IR cells (3076 cells in total) located in laminae I–III. The majority of HCN4-IR cells were found in laminae III (68.0%) and II_iv_ (26.3%), whereas there were only a few HCN4-IR cells in laminae I–II_id_ (5.7%; Fig. 2d). These HCN4-IR cells were not detectable in DOX-treated HCN4^Luc/tTA_TRE^ mice without any changes in the distribution of PKCγ-IR cells (Fig. 2b). All HCN4-IR cells were NeuN-immunoreactivity-positive, implying that HCN4 was exclusively expressed in neurons (Figs. 2, 4, Supplementary Fig. S1). Quantitative analysis revealed that HCN4-IR cells accounted for 1.7% (range 0–4.3%, *n* = 3 mice), 15.3% (14.3–17.0%), and 19.7% (17.0–22.8%) of all neurons in laminae I–II_id_, II_iv_, and III, respectively (Fig. 2c).

### Colocalization of HCN4-immunoreactivity with PV- or PKCγ-immunoreactivity

**Fig. 3:**
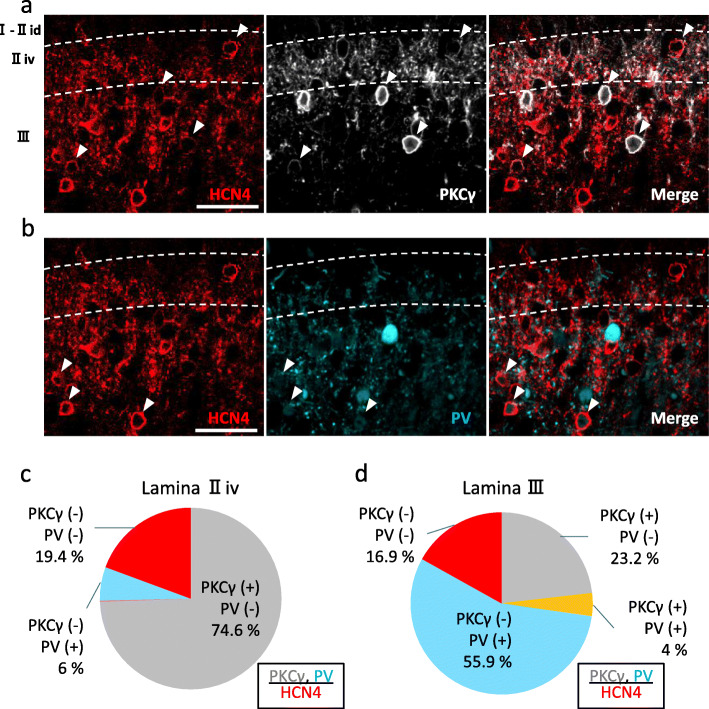
Colocalization of HCN4-immunoreactivity with PKCγ- or PV-immunoreactivity. **a** Colocalization of HCN4 and PKCγ. *Left panel*, immunohistochemistry for HCN4 (red). *Center panel*, PKCγ immunohistochemistry (white). *Right panel*, overlay of HCN4- and PKCγ-immunoreactivities. Arrowheads indicate cells IR for both HCN4 and PKCγ. **b** Colocalization of HCN4 and PV. *Left panel*, HCN4 immunohistochemistry (red). *Center panel*, immunohistochemistry for PV (cyan). *Right panel*, overlay of HCN4- and PV-immunoreactivities. Arrowheads indicate cells IR for both HCN4 and PV. Note that the PV-IR intensity in HCN4-IR cells is relatively weak. **a**, **b** Scale bars, 40 μm. Dashed lines indicate the borders of the PKCγ plexus. **c**, **d** Proportions of HCN4-IR cells in lamina II_iv_ (**c**) and III (**d**) that colocalize with PKCγ- and/or PV-immunoreactivities

In the murine SDH, 90–95% of the neurons in lamina I are interneurons. The great majority of neurons in laminae II and III are also interneurons [24, 25]. These interneurons show neurochemical diversity and are known to be allocated to subpopulations which can be defined by expression of specific markers [24, 26, 27]. Among these markers, we first examined the PV- and PKCγ-immunoreactivity in HCN4-IR cells. In lamina II_iv_, only 6.0% of HCN4-IR cells were PV-IR (Fig. 3b, c). By contrast, 59.9% of HCN4-IR cells in lamina III were PV-IR (Fig. 3b, d). We found most of these PV-IR signals in HCN4-IR cells were very weak, as shown in Fig. 3b.

PKCγ-IR cells accounted for 74.6% of HCN4-IR cells in lamina II_iv_ (Fig. 3a, d) and 27.2% in lamina III (Fig. 3d). Among HCN4-IR cells, 19.4% (lamina II_iv_) and 16.9% (lamina III) were neither PV-IR nor PKCγ-IR. These results seemed to support the findings that 78–84% of PV-IR cells and 53.9% of PKCγ-IR cells in laminae II–III are reportedly HCN4-IR [13, 17].

### Virtually all HCN4-IR cells in laminae II_iv_–III are excitatory interneurons

Reportedly, only ~ 70% of PV-IR cells in the murine SDH express VGAT mRNA. The rest of PV-IR cells (~ 30%) express mRNA of the vesicular glutamate transporter 2 (VGLUT2) [28]. PKCγ cells in this area constitute a subset of excitatory interneurons [24]. Thus, we asked next whether HCN4-IR cells are excitatory or inhibitory neurons because HCN4 channels were suggested to be expressed in ‘putative’ inhibitory interneurons expressing PV, in addition to excitatory PKCγ cells.

**Fig. 4:**
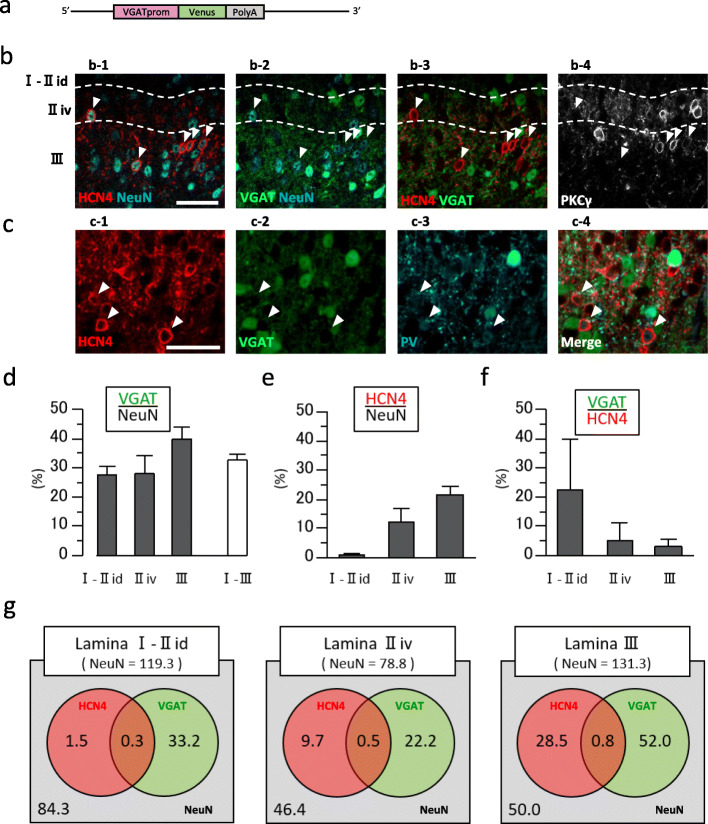
The majority of HCN4-IR cells do not express VGAT. **a** Schematic diagram of the recombinant allele in the VGAT-Venus BAC transgenic mouse. **b** Colocalization of HCN4-immunoreactivity and VGAT-Venus. **b-1** Immunoreactivity for HCN4 (red) and NeuN (cyan). **b-2** VGAT-Venus (green) and NeuN (cyan). **b-3** Overlay of HCN4-immunoreactivity (red) and VGAT-Venus (green). **b-4** PKCγ (gray). HCN4-IR cells indicated by arrowheads do not colocalize with Venus. **c** Colocalization of HCN4 (red), VGAT (green), and PV (cyan). **c-1** Immunoreactivity for HCN4 (red). **c-2** VGAT-Venus (green). **c-3** Immunoreactivity for PV (cyan). **c-4** Overlay of HCN4-immunoreactivity (red), VGAT-Venus (green), and PV-immunoreactivity (cyan). Arrowheads indicate HCN4- and PV-IR cells. Note that these cells do not colocalize with VGAT-Venus. It should be also noted that the intensity of PV-immunoreactivity in PV-IR and Venus-positive cells are higher than that in HCN4- and PV-IR cells. **b**, **c** Scale bars, 40 μm. The image was obtained in the same preparation shown in Fig. 3a, b. **d** Proportions of NeuN-IR cells that coexpress VGAT-Venus in each lamina. **e** Proportions of NeuN-IR cells that coexpress HCN4-immunoreactivity in each lamina. **f** Proportions of HCN4-IR cells that coexpress Venus in each lamina. **g** Venn diagrams showing the relationships among populations of HCN4-IR cells (red circle), VGAT-Venus-expressing cells (green circle), and NeuN-IR cells (gray rectangle) in each lamina. The number shown in each category indicates the average number of cells identified in an 8-μm thick slice

In the SDH, inhibitory interneurons are GABAergic and/or glycinergic; GABAergic neurons tend to be concentrated in superficial areas whereas glycinergic neurons occupy deeper laminae [24]. Because the VGAT is involved in transporting both GABA and glycine into presynaptic vesicles, we crossed HCN4^+/tTA_TRE^ mice with VGAT-Venus mice and aimed to determine the population of inhibitory HCN4-IR neurons using the double transgenic HCN4^+/tTA_TRE^::VGAT-Venus mice [21] (Fig. 4). Quantitative analyses of these mice revealed that Venus-expressing neurons accounted for 22.2% of HCN4-IR cells in laminae I–II_id_ (range 0–33.3%, *n* = 3 mice), 5.2% in lamina II_iv_ (0–11.8%), and 2.9% (0–4.8%) in lamina III. These findings confirmed that the majority of HCN4-IR cells were not inhibitory interneurons, but excitatory interneurons. Furthermore, most of the cells that were IR for both PV and HCN4 were not Venus-positive, suggesting that HCN4 was expressed by excitatory PV-IR cells.

In addition, we also carried out immunohistochemical studies in the double transgenic HCN4^+/tTA_TRE^::GAD67-GFP mouse in which GABAergic inhibitory neurons are visualized by GFP (Supplementary Fig. S1). Quantitative analyses revealed that GFP-expressing cells accounted for 3.3% (range 0–10.0%, *n* = 3 animals) of HCN4-IR cells in laminae I–II_id_, 4.0% (0–8.7%) in lamina II_iv_, and 4.1% (1.6–9.1%) in lamina III. We therefore concluded that the vast majority of HCN4-IR cells in laminae I–III were not inhibitory interneurons.

### HCN4-immunoreactivity of VGLUT2-IR presynaptic terminals

**Fig. 5:**
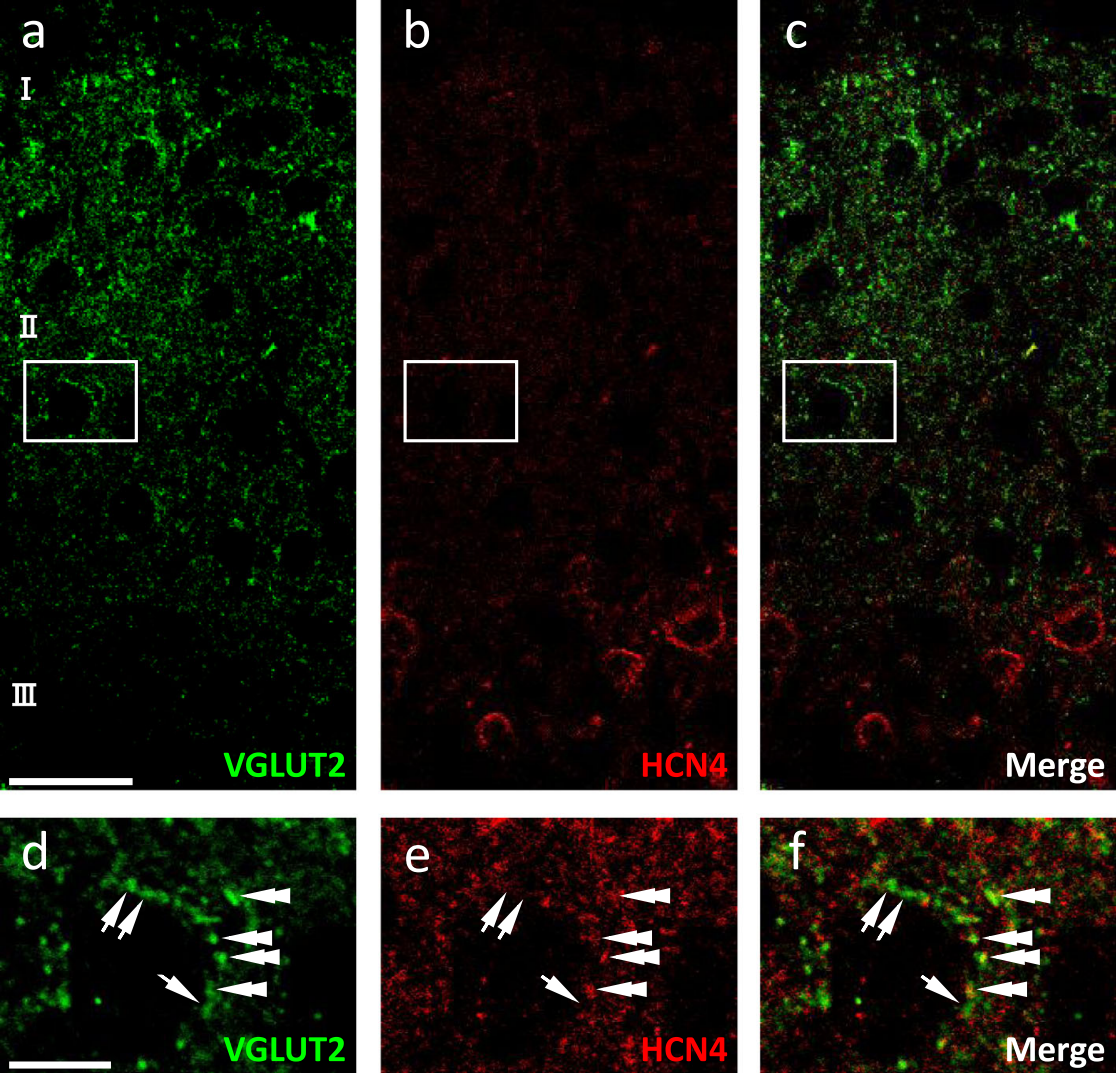
Colocalization of VGLUT2-immunoreactivity and HCN4-immunoreactivity. **a**, **d** Immunohistochemistry for VGLUT2 (green). **b**, **e** Immunohistochemistry for HCN4 (red). **c**, **f** Overlay of VGLUT2- and HCN4-immunoreactivities. Upper and lower panels are images at low and high magnifications, respectively. The areas indicated by the white boxes are expanded in lower panels. Arrowheads indicate VGLUT2-IR terminals that also show HCN4-immunostaining. Double arrowheads and arrows indicate VGLUT2-IR terminals with and without HCN4-immunostaining, respectively. Scale bars, 20 μm (upper panels) and 5 μm (lower panels)

In the present study, we observed punctate HCN4-IR signals in addition to circumferential membrane immunostaining. These findings strongly indicated that HCN4 was expressed both on neurites and the soma. Glutamate is transported into the synaptic vesicle by VGLUT2 in presynaptic terminals of spinal excitatory interneurons and some myelinated, but not most unmyelinated, primary afferent fibers [29]. Therefore, to examine whether HCN4 is expressed in presynaptic terminals of excitatory interneurons, we next examined the colocalization of HCN4-IR signals and VGLUT2-IR signals [14]. In good agreement with a previous report [29], the VGLUT2-IR signals showed punctate patterns corresponding to axonal varicosities and were widely distributed in the SDH (Fig. 5a). The punctate HCN4-IR signals were also detected in laminae I–III, although their density was lower in lamina II_o_ (Fig. 5b). At higher magnification, we found that some VGLUT2-IR terminals overlapped with punctate HCN4-IR signals (Fig. 5c-f). In this area, the VGLUT2-immunoreactivity represents terminals from primary afferents or excitatory interneurons. Among these terminals, the VGLUT2-immunoreactivity in those from unmyelinated afferents is weak while in those from interneurons are solid, and those from myelinated afferents forming synaptic glomeruli are larger than those from interneurons. Because some VGLUT2-IR terminals with HCN4-immunoreactivity were relatively small, our finding suggested that HCN4 was expressed in presynaptic terminals of a subset of excitatory interneurons [14, 24]. The punctate HCN4-IR signals that did not overlap with VGLUT2-immunoreactivity might be located on dendrites of excitatory interneurons.

### Most HCN4-IR neurons in the SDH did not respond to acute nociceptive stimulation

**Fig. 6:**
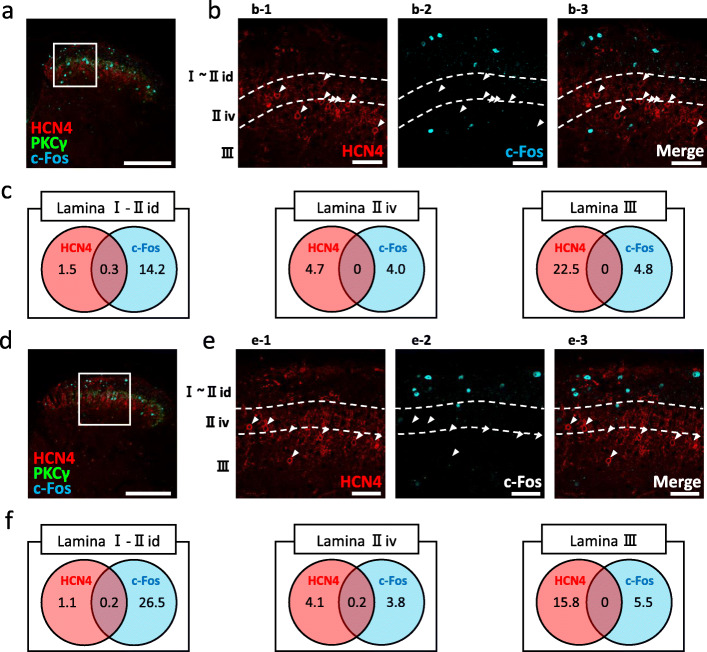
Nociceptive stimulation does not induce c-Fos in HCN4-IR cells. **a** Induction of c-Fos expression in the SDH by pinching. Immunoreactivities for HCN4 (red), PKCγ (green), and c-Fos (cyan). Scale bar, 200 μm. The area indicated by the white box is expanded in **b**. **b** Colocalization of HCN4- and c-Fos-immunoreactivities. **b-1** Immunoreactivity for HCN4. **b-2** Immunoreactivity for c-Fos. Note that c-Fos-immunoreactivity is mainly induced in lamina I. **b-3** Overlay of HCN4- and c-Fos-immunoreactivity. Scale bar, 40 μm. Dashed lines indicate the borders of the PKCγ plexus. **c** Venn diagrams showing the relationship between the populations of HCN4-IR cells (red circle) and c-Fos-IR cells (cyan circle) in each lamina. The number shown in each category indicates the average number of cells identified in an 8-μm thick slice. **d**-**f** Induction of c-Fos expression in the SDH by thermal nociceptive stimulation. Symbols and scale bars correspond to those in **a**-**c**

Next, we examined whether HCN4-IR cells can respond to acute nociceptive stimulation. The induction of Fos expression has been commonly used to identify activation of SDH neurons by noxious stimulation [30]. For this purpose, we applied mechanical (pinching the hind paw; Fig. 6a-c) or thermal (immersion of the hind paw into hot water; Fig. 6d-f) noxious stimuli.

After the pinch stimulation, we identified on average 14.5 c-Fos-IR cells per hemisection (6 hemisections in 3 animals) in laminae I–II_id_, 4.0 in lamina II_iv_, and 4.8 in lamina III. Neurons that showed both HCN4- and c-Fos-immunoreactivity accounted for 18.2% (range 0–50%) of HCN4-IR cells in laminae I–II_id_. In laminae II_iv_ and III, c-Fos expression was not induced in HCN4-IR cells (Fig. 6c).

Following thermal noxious stimulation, we identified an average of 26.7 c-Fos-IR cells per hemisection (6 hemisections in 3 animals) in laminae I–II_id_, 4.0 in lamina II_iv_, and 5.5 in lamina III. The neurons that showed both HCN4- and c-Fos-immunoreactivity accounted for 12.5% (range 0–25%) of HCN4-IR cells in laminae I–II_id_. The neurons that showed both HCN4- and c-Fos-immunoreactivity accounted for 3.8% (range 0–8.3%) of HCN4-IR cells in lamina II_iv_. No HCN4-IR cell in lamina III expressed c-Fos (Fig. 6f). These results suggested that the majority of HCN4-IR cells in the SDH were not directly involved in acute nociception.

As shown in Fig. 1c, the expression of HCN4 was completely suppressed in HCN4^Luc/tTA_TRE^ mice with DOX administration (HCN4-knockdown mice). Two weeks after starting DOX administration, we could not observe obvious alteration in general behavior of HCN4-knockdown mice. This finding was in good agreement with the previous report on brain-specific HCN4 conditional knockout mice [31]. We then examined the nocifensive behavior in HCN4-knockdown mice using von Frey assay. We could not find significant difference in the withdrawal threshold between WT and HCN4-knock down mice, as shown in supplementary Fig. S2. This finding seems to support the finding that c-Fos expression was not induced in the HCN4-positive cells by nociceptive stimulation.

## Discussion

In this study, we described for the first time the distribution of HCN4-immunoreactivity in the mouse spinal cord by immunohistochemistry using a knockdown-verified specific antibody. We found that HCN4-immunoreactivity was concentrated in the SDH, especially near the border of laminae II and III. We also examined the colocalization of HCN4-immunoreactivity with commonly used neurochemical markers and revealed that most HCN4-IR cells expressed either PKCγ or PV. Using transgenic mice in which fluorescent reporter proteins are selectively expressed only in inhibitory neurons, we demonstrated for the first time that the vast majority of HCN4-IR cells were limited to excitatory interneurons in this area.

In our previous report, we verified the specificity of the same antibody against HCN4 by western blotting of protein samples obtained from the same set of HCN4-transgenic mice used in this study. It is not known, however, whether an antibody whose specificity has been verified by western blotting can provide specific immunohistochemical staining of brain/spinal cord slice preparations in general. To verify whether immunoreactivities with the same antibody were specific against HCN4 in immunohistochemistry, we knocked down the expression of HCN4 in HCN4^Luc/tTA_TRE^ mice by DOX administration. Consistent with our previous western blotting experiments, HCN4-immunoreactivity was completely absent following DOX treatment. This result allows us to consider that HCN4-immunoreactivity faithfully reflects the locations of HCN4 proteins in the spinal cord. In other studies, HCN4-immunoreactivities were reportedly found throughout the spinal cord including the ventral horn [17]. However, the antibodies used in those studies were not verified with HCN4-knockout or -knockdown mice, and a tyramide signal amplification method was necessary to visualize HCN4-immunoreactivity in neurites and somata [13, 17]. As demonstrated in the present study, we did not observe HCN4-IR somata in the ventral horn even in tissues obtained from HCN4^+/tTA_TRE^ mice, in which HCN4 was overexpressed at its physiological expression loci. This result also agreed with the finding that in HCN4^Luc/tTA_TRE^ mice, the luminescence signals were restricted to the SDH. Based on these findings, we concluded that the location of HCN4-IR neurons was restricted to the SDH.

### Expression of HCN4 in excitatory PV-neurons

In this study, we confirmed that 60% of the HCN4-IR neurons were PV-IR in lamina III (Fig. 3). In accordance with our findings, Hughes et al. (2012) also reported that 77.9% of PV-IR cells in laminae II_i_–III showed HCN4-immunoreactivity, whereas 64.8% of them were HCN1-IR [13].

In the SDH, the population of PV-positive neurons reportedly consists of both inhibitory (70%) and excitatory (30%) interneurons [28, 32]. It has been reported that PV-expressing inhibitory interneurons in laminae II_i_–III are the source of axo-axonic inputs onto presynaptic terminals of low-threshold mechanoreceptor (LTMR) afferents and exert presynaptic inhibition. PV-terminals having contacts with LTMR afferents were clearly shown to be VGAT-positive, and the dysfunction of inhibitory PV-neurons was implicated in the development of tactile allodynia [13, 33, 34]. In lamina II, inhibitory neurons reportedly tend to have tonic firing patterns. These inhibitory neurons sustain high-frequency firing, which is often associated with the presence of the I_h_ current.

Seemingly, this rule does not apply to the neurons in LTMR recipient zone (LTMR-RZ). In the LTMR-RZ, many types of neurons show tonic firing patterns. Most notably, both excitatory and inhibitory PV-neurons in the LTMR-RZ exhibit tonic firing and the voltage sag (a slowly developing depolarization during hyperpolarizing current injection), which is generated by the activation of I_h_ upon hyperpolarization [28]. Although Tae et al. (2017) recorded I_h_ in putative inhibitory neurons that expressed GFP driven by the PV promoter, they also demonstrated that Pax2 (a marker of inhibitory neurons) did not colocalize with HCN4 [35]. Furthermore, we found that almost all HCN4-IR neurons lacked fluorescent proteins in VGAT-Venus or GAD67-GFP mice (Fig. 4, Supplementary Fig. S1). Therefore, our data provided for the first time strong evidence that HCN4- and PV-IR neurons were excitatory interneurons.

### Expression of HCN4 in excitatory PKCγ-neurons

Another population of neurons in which we observed HCN4-immunoreactivity in laminae II_iv_ and III was that of PKCγ-IR neurons (Fig. 3). In accordance with our observation, previous studies reported that HCN4-immunolabeling was found in 53.9% of PKCγ-IR cells in the SDH [17]. Moreover, I_h_ was recorded in 70.8% of PKCγ-positive interneurons [36]. However, in another study, PKCγ-positive neurons only showed a very limited voltage sag upon hyperpolarization, whereas cholecystokinin (CCK)-positive neurons displayed a small but detectable voltage sag, suggesting the presence of a sizable I_h_ current [28]. It appears a matter of debate whether CCK-expressing neurons are a subpopulation of PKCγ neurons or an independent population that does not express PKCγ [37, 38]. In the former case, HCN4-expressing PKCγ-IR cells might be a CCK-expressing subpopulation of PKCγ cells. In the latter case, CCK-positive neurons might correspond to HCN4-IR cells that were neither PKCγ-IR nor PV-IR (Fig. 3).

Peripheral information is conveyed by primary afferent fibers. In the SDH, they are not only relayed to projection neurons but also processed by local circuits. Recently, genetic ablation studies of spinal interneurons have revealed the importance of such local circuits for the transmission of noxious, pruritic, and tactile sensations [39]. It is established that PKCγ is expressed by excitatory interneurons [15, 16]. In the local circuit of lamina II, noxious unmyelinated fibers form synaptic connections with transient central (TC) neurons. TC neurons form monosynaptic excitatory connections to vertical neurons in lamina II_o_, which successively have monosynaptic excitatory connections with nociceptive projection neurons in lamina I [40]. Besides, innocuous myelinated afferent fibers reportedly connect to PKCγ-IR interneurons in laminae II_iv_–III [41]. Although PKCγ-IR interneurons also form monosynaptic excitatory connections with nociceptive TC neurons, Aβ-fiber inputs simultaneously activate glycinergic inhibitory interneurons, which suppress PKCγ-IR interneurons. This feed-forward inhibitory mechanism was suggested to prevent the tactile Aβ-input from activating the nociceptive pathway.

It has been suggested that peripheral nerve injury suppresses this feed-forward mechanism and induces mechanical allodynia [42]. In the PKCγ-knockout mouse, tactile allodynia is attenuated without changing the response to acute pain [43]. Pharmacological inhibition of PKCγ also exerts similar effects [44]. These findings suggest that the remodeling of local circuits involving PKCγ-expressing neurons in the dorsal horn may be induced by peripheral nerve injury. As discussed above, HCN4-expressing PKCγ cells might be a CCK-expressing subpopulation of PKCγ cells. Interestingly, ablation studies of these cells demonstrate their contribution to texture discrimination in naïve animals [28], as well as their involvement in mechanical allodynia, which is determined by corticospinal projections [45]. Based on these studies, Gutierrez-Mecinas et al. (2019) suggested that CCK^+^/PKCγ^+^ neurons may be particularly important for the development of mechanical allodynia [37].

### Possible physiological roles of HCN4 channels in excitatory SDH interneurons

As shown in the present study, HCN4-expressing cells in the SDH did not respond to the noxious stimulation. This result seems to agree with the distribution of HCN4-expressing cells revealed in this study. The HCN4-expressing neurons largely overlapped with the PKCγ-expressing neurons in lamina II_iv_ ~ III. The PKCγ-expressing neurons reportedly receive the synaptic inputs from myelinated Aβ- and Aδ fibers, which convey tactile information, but not acute nociceptive information [41, 46]. However, in the mechanical allodynia, peripheral nerve injury has been suggested to induce the crosstalk from innocuous tactile circuitry to pain transmission circuitry. Most notably, the PKCγ-expressing neurons are reportedly one of components constructing the circuit to relay tactile inputs from the low-threshold mechanoreceptors to projection neurons processing noxious information [33, 41]. It has been also reported that the transgenic mice lacking PKCγ failed to develop mechanical allodynia induced by sciatic nerve injury [43]. These finding suggest that HCN4 channels expressed in PKCγ-positive cells may be involved in mechanical allodynia.

In PKCγ-expressing neurons of the rat SDH, the phosphorylation of cAMP response element-binding protein was reportedly increased after partial sciatic nerve ligation, suggesting that the ligation increased cAMP levels in PKCγ-expressing neurons [47]. Because intracellular cAMP reportedly activates HCN2 and HCN4 channels by shifting the voltage dependence of the activation curve [1], HCN4 channels expressed in PKCγ-IR interneurons may be activated by partial sciatic nerve ligation via an increase in cAMP. Peng et al. (2017) reported that HCN4-immunoreactivity was observed on presynaptic terminals connecting to putative excitatory interneurons and that pharmacological inhibition of HCN channels by ZD7288 decreased the frequency of miniature excitatory postsynaptic potentials [14]. These findings suggest that the constitutive activation of HCN4 channels may contribute to the resting membrane potential of presynaptic terminals. Therefore, it is speculated that presynaptic HCN4 channels may facilitate excitatory synaptic transmission if cAMP levels in presynaptic terminals are elevated under pathological conditions. Furthermore, it has been reported that expression levels of HCN channels in visceral organs are dynamically changed under pathological conditions. For example, HCN2 and HCN4 channels are upregulated in heart failure, giving rise to potentially lethal arrhythmias [48, 49]. Because the first intron of the *Hcn4* gene contains an enhancer element that is regulated by AP1 and MEF2 [50, 51], the expression of HCN4 in the SDH might be upregulated after nerve injury. We have also reported that HCN4 channels in the sinoatrial node attenuate the hyperpolarization induced by parasympathetic stimulation [19]. For the same rationale, HCN channels may antagonize the hyperpolarization of PKCγ-IR interneurons induced by the activity of inhibitory interneurons. HCN channels expressed in the spinal cord, as well as in the PNS, may be potential therapeutic targets of allodynia [35, 52, 53].

In the present study, global knockdown of HCN4 did not induced significant change in nocifensive behavior. However, it appears difficult to interpret the effects of global HCN4-knockdown, because HCN4 is widely expressed in many locus of the brain, and the pharmacological inhibition of I_h_ in the anterior cingulate cortex reportedly reduces mechanical allodynia [54]. In the future study, specific inhibition of I_h_ in the HCN4-expressing excitatory SDH interneurons may provide a clue to clarify the pathophysiological roles of these cells in mechanical allodynia.

## Supplementary information

**Additional file 1: Table S1.** Primary antibodies. **Table S2.** Secondary antibodies.

**Additional file 2: Figure S1.** The majority of HCN4-IR cells do not express GAD67.

**Additional file 3: Figure S2.** Fifty-percent paw-withdrawal threshold did not differ between wild type mice and HCN4-knockdown mice.

## Data Availability

All original data are available upon reasonable request. The transgenic mice used in this study are available at RIKEN BRC.

## Abbreviations

BAC: Bacterial artificial chromosome
cAMP: Cyclic adenosine monophosphate
CCK: Cholecystokinin
CNS: Central nervous system
DOX: Doxycycline
DRG: Dorsal root ganglion
GABA: Gamma-aminobutyric acid
GAD67: Glutamate decarboxylase 67
HCN: Hyperpolarization-activated, cyclic nucleotide-gated
I_h_: Hyperpolarization-activated, non-selective cation current
II_id_: Dorsal part of the inner lamina II
II_iv_: Ventral part of the inner lamina II
IR: Immunoreactive
LTMR-RZ: Low-threshold mechanoreceptor-recipient zone
PKCγ: Protein kinase Cγ
PNS: Peripheral nervous system
PV: Parvalbumin
qPCR: Quantitative real-time polymerase chain reaction
SDH: Spinal dorsal horn
TC: Transient central
tTA: Tetracycline transactivator
VGAT: Vesicular GABA transporter
VGLUT2: Vesicular glutamate transporter 2
