# Supplementary material for: Expression of the pacemaker channel HCN4 in excitatory interneurons in the dorsal horn of the murine spinal cord
Source: Mol Brain. 2020 Sep 18;13:127. doi: 10.1186/s13041-020-00666-6 (PMC7501643; doi:10.1186/s13041-020-00666-6)
Supplement: Supplementary file 1 — Additional file 1: Table S1. Primary antibodies. Table S2. Secondary antibodies. [file 13041_2020_666_MOESM1_ESM.docx]

| **Supplementary Table S1. Primary antibodies** | | | | |
| --- | --- | --- | --- | --- |
| Antigen | Host | Dilution | Cat. No | Sourse |
| c-Fos | Guinea Pig polyclonal | 1:500 | 226 004 | Synaptic Systems |
|  |  |  |  | Göttingen, Germany |
| HCN4 | Rabbit polyclonal | 1:1000 | APC-052 | Alomone Labs, |
|  |  |  |  | Jerusalem, Israel |
| HCN4 | Guinea Pig polyclonal | 1:1000 | AGP-004 | Alomone Labs, |
|  |  |  |  | Jerusalem, Israel |
| NeuN | mouse monoclonal | 1:1000 | MAB377 | Merck KGaA Chemicon, |
|  |  |  |  | Darmstadt, Germany |
| PKCγ | Guinea Pig polyclonal | 1:200 | AB_2571826 | Frontier Institute Co. Ltd |
|  |  |  |  | Hokkaido, Japan |
| PKCγ | Goat polyclonal | 1:200 | AB_2571825 | Frontier Institute Co. Ltd |
|  |  |  |  | Hokkaido, Japan |
| PV | Guinea Pig polyclonal | 1:500 | AB_2571615 | Frontier Institute Co. Ltd |
|  |  |  |  | Hokkaido, Japan |
| PV | Goat polyclonal | 1:500 | AB_2571614 | Frontier Institute Co. Ltd |
|  |  |  |  | Hokkaido, Japan |
| VGLUT2 | Rabbit polyclonal | 1:100 | bs-9686R | Bioss Antibodies |
|  |  |  |  | USA |

| **Supplementary Table 2. Secondary antibodies** | | | | | |
| --- | --- | --- | --- | --- | --- |
|  |  |  | Fluophore/ |  |  |
| Antigen | Species | Dilution | conjugate | Source | Cat. No |
| Goat | Donkey | 1:200 | DyLight 405 | Jackson Immunoresearch | 705-475-147 |
| Goat | Donkey | 1:200 | Alexa 594 | Jackson Immunoresearch | 705-586-147 |
| Guinea Pig | Donkey | 1:200 | Alexa 488 | Jackson Immunoresearch | 706-545-148 |
| Guinea Pig | Donkey | 1:500 | Alexa 647 | Jackson Immunoresearch | 706-605-148 |
| mouse | Donkey | 1:500 | DyLight 405 | Jackson Immunoresearch | 715-475-151 |
| Rabbit | Donkey | 1:50 | Alexa 488 | Invitrogen | R37118 |
| Rabbit | Donkey | 1:200 | Alexa 594 | Invitrogen | R37119 |
