# Supplementary material for: Expression of the pacemaker channel HCN4 in excitatory interneurons in the dorsal horn of the murine spinal cord
Source: Mol Brain. 2020 Sep 18;13:127. doi: 10.1186/s13041-020-00666-6 (PMC7501643; doi:10.1186/s13041-020-00666-6)
Supplement: Supplementary file 2 — Additional file 2: Figure S1. The majority of HCN4-IR cells do not express GAD67. [file 13041_2020_666_MOESM2_ESM.pdf]

Supplementary Figure S1. The majority of HCN4-IR cells do not express GAD67.

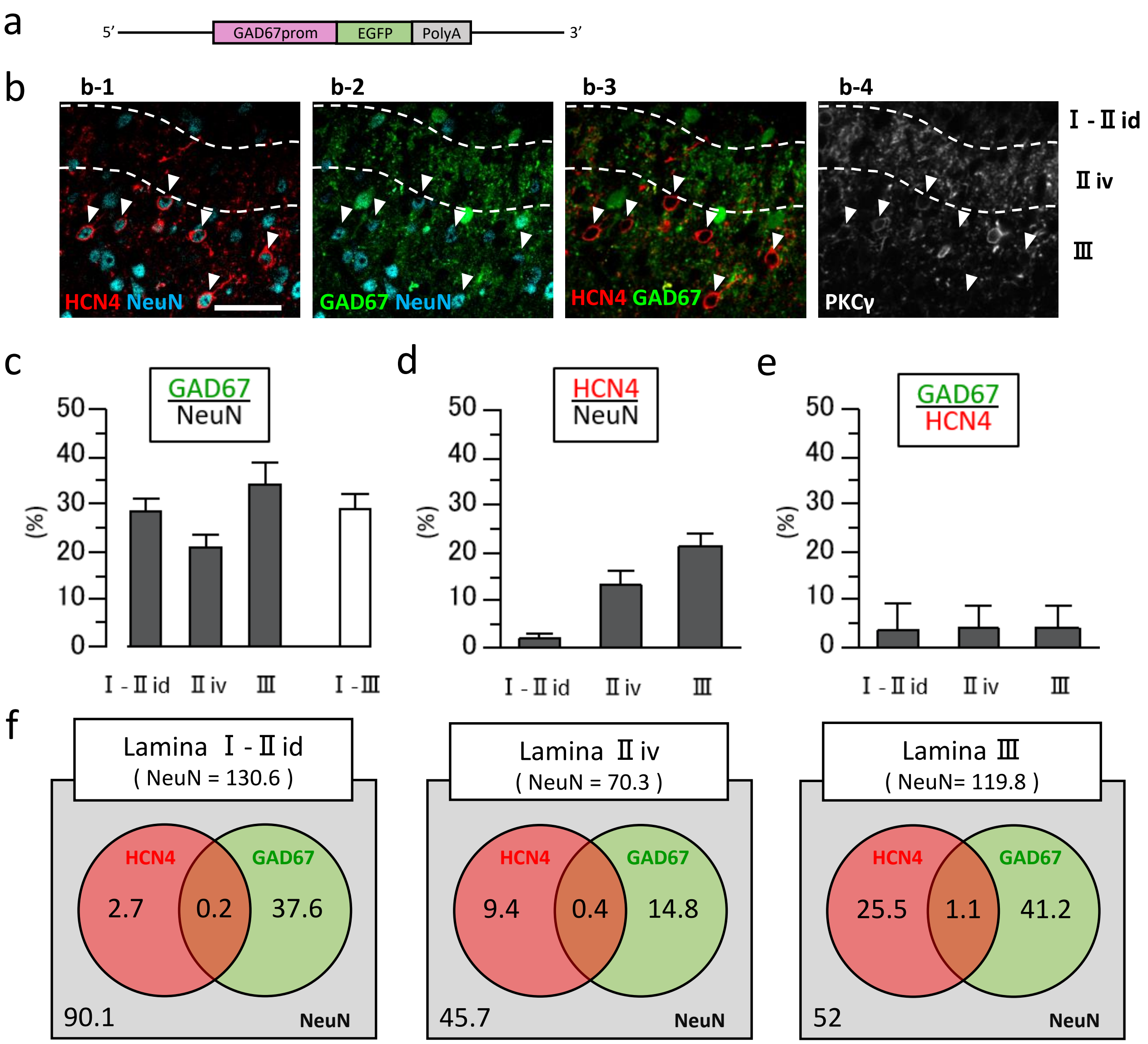

a Schematic diagram of the recombinant allele in the GAD67-GFP knock-in mouse. **b** Colocalization of HCN4-immunoreactivity and GAD67-GFP. **b-1** Immunoreactivity for HCN4 (red) and NeuN (cyan). **b-2** GAD67 -GFP (green) and NeuN (cyan). **b-3** Overlay of HCN4-immunoreactivity (red) and GAD67-GFP (green). **b-4** PKCγ (gray). HCN4-IR cells indicated by arrowheads do not colocalize with GFP. Scale bar, 40 µm. **c** Proportions of NeuN-IR cells that coexpress GAD67-GFP in each lamina. **d** Proportions of NeuN-IR cells that coexpress HCN4-immunoreactivity in each lamina. **e** Proportions of HCN4-IR cells that coexpress GAD67-GFP in each lamina. **f** Venn diagrams showing the relationships among populations of HCN4-IR cells (red circle), GAD67-GFP-expressing cells (green circle), and NeuN-IR cells (gray rectangle) in each lamina. The number shown in each category indicates the average number of cells identified in an 8-µm thick slice.
