# Supplementary material for: Expression of the pacemaker channel HCN4 in excitatory interneurons in the dorsal horn of the murine spinal cord
Source: Mol Brain. 2020 Sep 18;13:127. doi: 10.1186/s13041-020-00666-6 (PMC7501643; doi:10.1186/s13041-020-00666-6)
Supplement: Supplementary file 3 — Additional file 3: Figure S2. Fifty-percent paw-withdrawal threshold did not differ between wild type mice and HCN4-knockdown mice. [file 13041_2020_666_MOESM3_ESM.pdf]

Supplementary Figure S2. Fifty-percent paw-withdrawal threshold did not differ between wild type mice and HCN4-knockdown mice.

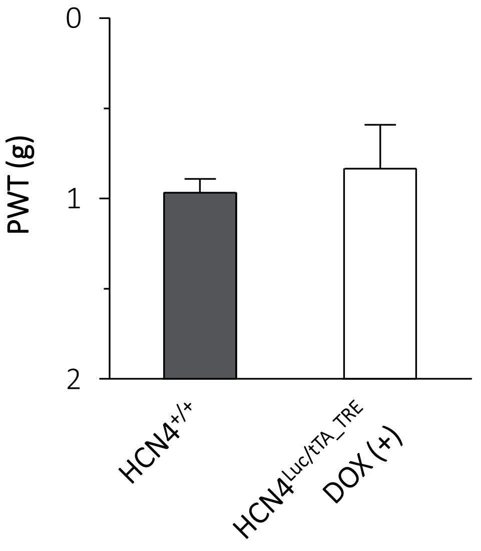

Fifty-percent paw-withdrawal thresholds to von Frey fiber stimulation in wild type mice (n = 3) and $HCN4^{Luc/tTA\_TRE}$ mice with DOX administration (n = 3) by up-down method (p = 0.40, Student’s unpaired t test).
